# Thermal decomposition study of 4-methyloxybenzyl-glycoside by TG/DTA and on-line pyrolysis-photoionization mass spectrometry

**DOI:** 10.1038/s41598-024-62734-4

**Published:** 2024-05-24

**Authors:** Lei Wu, Yiming Wang, Liutianyi Yang, Meiling Jian, Yu Ding

**Affiliations:** 1 Flavour and Fragrance Department, Sichuan Sanlian New Materials Co. Ltd., Chengdu, 610101 China; 2 Harmful Components and Tar Reduction in Cigarette Sichuan Key Laboratory, Chengdu, 610066 China

**Keywords:** Aromatic alcoholic glycoside, Flavor precursor, Thermal decomposition, Photoionization mass spectrometry, Chemistry, Optics and photonics

## Abstract

A flavor precursor of 4-methyloxybenzyl-2, 3, 4, 6-tetra-O-acetyl-β-d-glucopyranoside (MBGL) was synthesized via a modified Koenigs–Knorr reaction. The thermal decomposition behaviour and pyrolysis intermediate products of the glycoside were studied by simultaneous thermogravimetric/differential thermal analysis (TG/DTA) and synchrotron vacuum ultraviolet (VUV) photoionization mass spectrometry (PIMS). TG/DTA results showed that the largest mass loss rate appeared at a *T*_*p*_ of 246.7 °C. PIMS was used to identify the pyrolysis products of MBGL at 300 °C, 500 °C and 700 °C, respectively. The experimental apparatus had some advantages in real-time analysis and fewer secondary reactions. Some important pyrolysis intermediates, such as the ions of the 4-methyloxybenzyl group at *m/z* 121 and the glycone moiety at *m/z* 347, were detected by PIMS. The results indicate that the MBGL was probably showed a different pyrolysis way compared with the other glycosides. This work reports a useful application of synchrotron VUV PIMS in a thermal decomposition study of glycoside flavor precursors.

## Introduction

Glycosides used as flavor precursors have been studied extensively. In tea leaves, many kinds of alcohols such as linalool and geraniol are reportedly present as glycosides. These aroma compounds can be liberated from their glycosidic flavor precursors by the action of endogenous enzymes during the tea manufacturing process^1–3^. In practice, glycosidic flavor precursors are common in many plant materials^4,5^. It is clear that flavorless glycosides represent one accumulation form of aroma substances in plant tissue^6^. Although these glycosides are flavorless and non-volatile, their hydrolysis may release aroma compounds that play an important role in the flavor of plants^7^.

The rupture of the O-glycosidic bond can be carried out by hydrolysis or by pyrolysis. In a previous study, to examine the potential use of glycosides as additives in high-temperature processing, glycosides with different aglycones were synthesized^8,9^. Thermogravimetric/differential thermal analysis (TG/DTA)^10,11^ and/or pyrolysis–gas chromatography–mass spectrometry (Py–GC/MS)^12–14^ were used to investigate the pyrolysis process and its decomposition products. However, it is well-known that the above techniques have some drawbacks in the study of the pyrolysis process, such as problems with real-time analysis and secondary reaction. During the thermal decomposition process, the primary cleavage of bonds is usually followed by a complex series of competitive reactions between the radicals and the fragment molecules. These secondary reactions complicate the pyrolysis products and make it difficult to clearly understand the mechanism of the decomposition process.

Anisyl alcohol is an important compound in the flavouring industry. However, its volatility limits its wider application. In our work, a glycosidic flavor precursor of 4-methyloxybenzyl-2, 3, 4, 6-tetra-O-acetyl-β-d-glucopyranoside (MBGL) was stereospecifically synthesized. Then, synchrotron vacuum ultraviolet (VUV) photoionization combined with molecular-beam mass spectrometry (also called photoionization mass spectrometry, PIMS) was used to investigate its thermal decomposition products. The experimental apparatus offered some advantages for analyzing the pyrolytic process of the glycoside flavor precursor, such as real-time analysis and fewer secondary reactions. Offering a wide tunability in the VUV region, synchrotron VUV photoionization mass spectrometry can minimize fragmentation and detect radicals. The principal thermal degradation intermediates of MBGL were investigated using PIMS at different temperatures. The results indicated that MBGL probably exhibited a different pyrolysis method than do other glycosides.

## Experimental section

### Glycoside preparation

**Figure 1:**
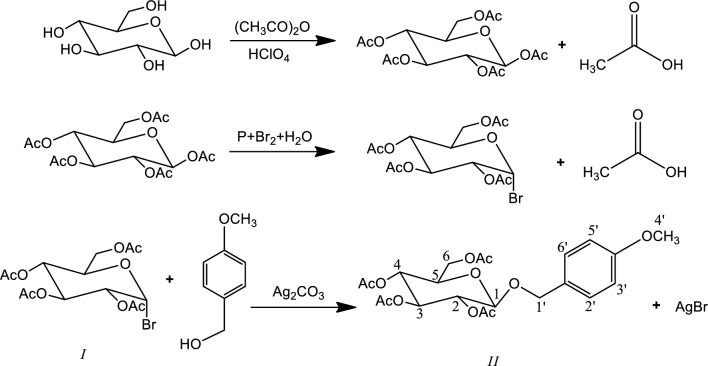
Gerneral synthetic process for MBGL.

In our study, MBGL was synthesized using the modified Koenigs–Knorr reaction^15^ under strictly anhydrous conditions (Fig. 1). All chemical reagents used in our study were of analytical grade and were purchased from Sinopharm Chemical Reagent Co. (Shanghai, China).

A detailed description of our method can be found in the literature^17^. First, acetobromo-α-D-glucose (2, 3, 4, 6-tetra-O-acetyl-α-d-glucopyranosyl bromide, *I* in Fig. 1) was prepared in 40 mL acetic anhydride via the successive addition of 10 g dry glucose, 3.1 g red phosphorous, 6 mL liquid bromine, and 3.6 mL water. At the end of the reaction, 30 mL dichloromethane and 2 × 80 mL ice water were added to the mixture. The mixture was vigorously shaken, and the phases were allowed to separate. The upper aqueous phase was discarded. Then, 80 mL of saturated sodium bicarbonate solution was added, and the same extraction process was repeated. The acetobromo-α-D-glucose was eventually retained in the dichloromethane. Then, 2.07 g of 4-methyloxy alcohol and 5 g of 4 Å molecular sieve were mixed with acetobromo-α-D-glucose dispersed in 80 mL of dichloromethane. Finally, 4.14 g of freshly prepared, dried silver carbonate was added to the mixture. The reaction mixture was kept in the dark and stirred for approximately 48 h. On completion of the reaction, the mixture was filtered, condensed, and separated by silica gel column chromatography. Pure products were obtained.

### Glycoside characterization

To identify the structure of the glycoside, its IR spectra and ^1^HNMR spectra were recorded. In this work, IR spectra (wave number range 4000–400 cm^−1^) were recorded with an EQUINOX55 infrared spectrometer (Bruker, Germany). The resolution of the instrument was 2.0 cm^−1^. The sample was analyzed as KBr micropellets. The software Origin 7.5 was used for processing of the IR data exported in TXT format. ^1^HNMR spectral data were obtained on a BRUKER AVANCE 300 MHz spectrometer with CDCl_3_ as the solvent and TMS as the internal reference standard. After exporting the original data file, the software Bruker Topspin 2.0 was used for further processing of the ^1^HNMR data.

### Thermoanalysis of TG/DTA

TG/DTA were carried out using a DTG-60H thermal analyzer (Shimadzu, Japan) with a 50 µL of aluminum crucible. TG sensitivity 0.1 µg, DTA sensitivity 0.01 µV. The temperature was programmed from ambient to 500 °C with a heating rate of 10 °C/min in air. The sample amount was 4.40 mg. The software TA-60WS was used to process the TG/DTA data. The software Origin 7.5 was used for visualizing the TG/DTA data.

### Glycoside pyrolysis

Pyrolysis was performed at the National Synchrotron Radiation Laboratory in Hefei, China. A detailed description of the instrument setup can be obtained from the literature^16^, and only a brief description is presented here. The thermal decomposition apparatus comprised a pyrolysis chamber equipped with a Shimadzu pyrolyzer (PYR-2A, Nakagyo-ku, Kyoto, Japan) and a photoionization chamber equipped with a reflectron time-of-flight mass spectrometer (approximate mass resolving power of 1400)^17^. The available photon energy range covered 7.8–24.0 eV with an average photon flux exceeding 10^13^ photons/s. Argon was used as the carrier gas.

During the experiment, the furnace was heated to a specific temperature, and approximately 50 mg of sample inside a scoop with a stainless steel pole of 6 mm diameter was pushed into the furnace. The species produced during the thermal decomposition process absorbed VUV photons, and were then ionized. The experiment was performed out at low pressure to prevent secondary reactions. With this “soft” photoionization method, the pyrolysis products were detected as molecular ions, M^+^. A series of mass spectra were collected at different pyrolysis temperatures and photon energies.

## Results and discussion

### Glycosides characterization

The structure of the synthetic glycoside was confirmed by its IR and ^1^HNMR spectroscopy. From the results, we could see that the IR and ^1^HNMR data perfectly corresponded to the molecular structure of the MBGL (see Table 1). The yield of MBGL was 53.0% (purity 99.2%).

**Table 1:** ^1^HNMR and IR identification of the synthesized MBGL.

Methods	Data	Structure
^1^HNMR, δ in CDCl_3_)	1.99–2.10 (12H, C-7, 8, 9, 10, CH_3_)	
	3.68 (1H, C-5, CH)	
	3.80 (3H, C-4′, CH_3_)	
	4.16–4.30 (2H, C-6, CH_2_)	
	4.50–4.53 (1H, C-1, CH)	
	4.56–4.83 (2H, C-1′, CH_2_)	
	5.03–5.20 (3H, C-2, 3, 4, CH)	
	6.80 (2H, C-3′, 5′, CH)	
	7.40 (2H, C-2′, 6′, CH)	
IR/cm^−1^, (KBr micropellets)	3013 (benzene ring, stretching vibration of C–H bond)	
	2945 (CH_3_, stretching vibration of C–H bond)	
	1752 (stretching vibration of C=O bond)	
	1499, 1453, 1428 (vibration of benzene ring)	
	1150–1070 (stretching vibration of C–O–C bond)	

### Thermoanalysis of MBGL

**Figure 2:**
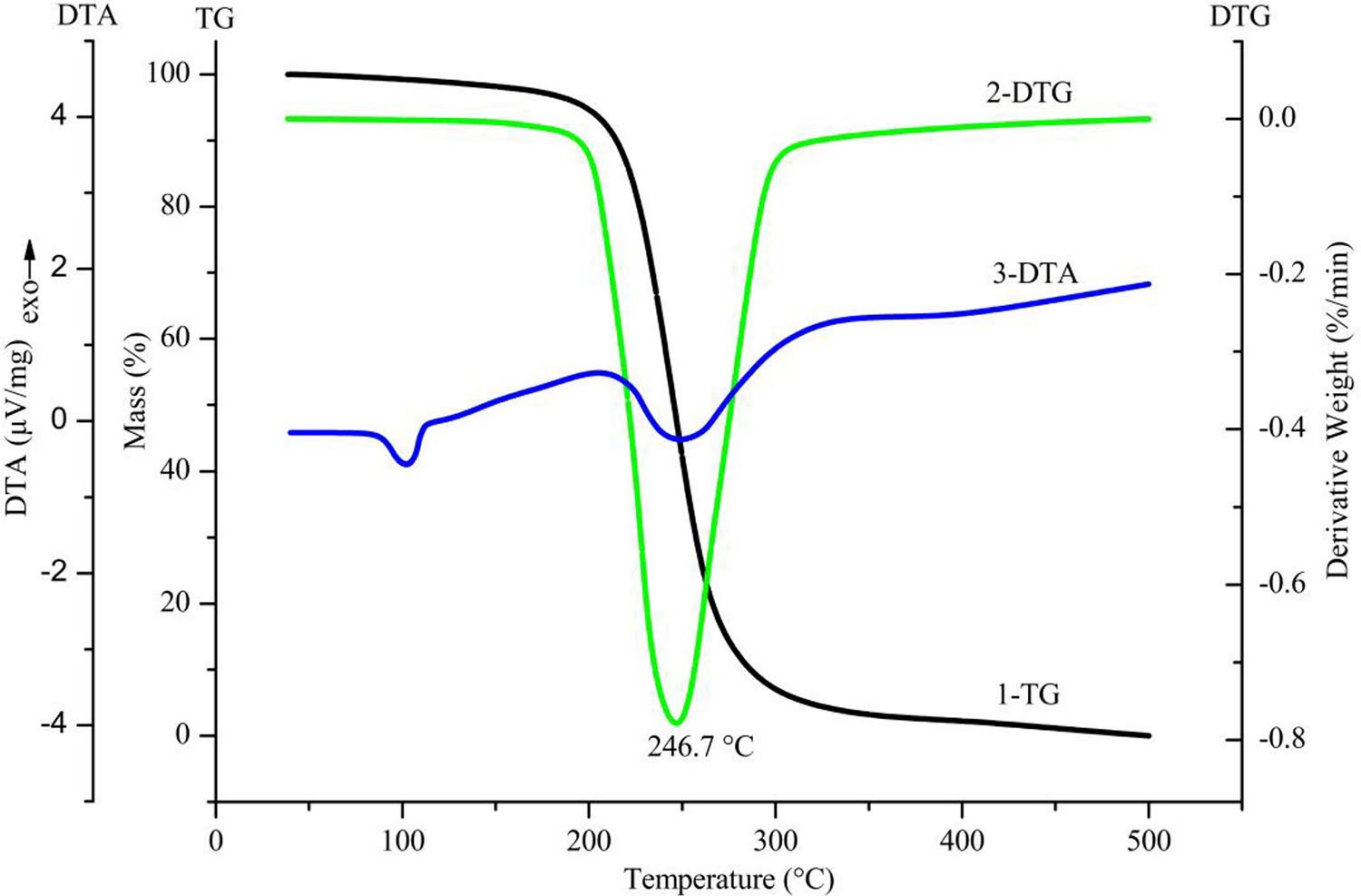
The TG–DTA curves of MBGL.

Figure 2 shows both TG and DTA curves recorded for the MBGL against temperature from room temperature to 500 °C. The TG–DTG curves reveal the relationship between the temperature change and mass loss of the MBGL. It was observed that the thermal decomposition of the MBGL mainly occurred between 200 and 300 °C. The largest mass loss rate appeared at a *T*_*p*_ of 246.7 °C with a total mass loss of 97.8% overall. A small mass loss between approximately 100 °C and 200 °C may be caused by sample condensation or deacetylation, according to the literature^9^. The DTA curve reveals the pyrolysis process of MBGL included two endothermic peaks. The first peak at approximately 101 °C may be attributed to the phase transition of the sample. The second peak at approximately 247 °C was the main one that was caused by the degradation of the sample.

### Selection of the pyrolysis temperature and photon energy

The pyrolysis temperatures were selected considering the onset decomposition temperature of MBGL. The above TG/DTA analysis results revealed that the thermal decomposition of the MBGL mainly occurred between 200 and 300 °C. The pyrolysis temperatures of 300 °C, 500 °C and 700 °C were chosen.

Benefiting from the wide tunability in the VUV region, a series of mass spectra were obtained at a fixed temperature by varying the photon energy. The results showed that the photon energy range from 9.0 to 13.5 eV was optimal. In this range, the pyrolysis products of MBGL were ionized well. As the photon energy increased to 14.5 eV, more fragment ion peaks were recorded in the mass spectra (see Fig. S1). Finally, a photon energy of 13.5 eV was chosen.

### Pyrolysis of MBGL

The TG/DTA analysis results showed that the MBGL had good thermal stability. However, the pyrolysis products were not identified. The identification of thermal decomposition products, especially some intermediates, is important for a better understanding of the pyrolysis mechanism of MBGL. Benefiting from the “soft” photoionization method mentioned above and fewer secondary reactions at low pressure, the pyrolysis products were detected as molecular ions, M^+^^16^.

**Figure 3:**
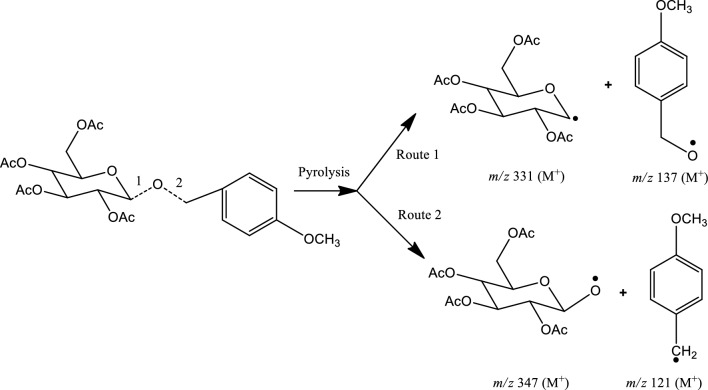
Two possible pyrolysis modes for MBGL.

It is well known that the primary decomposition reaction of glycoside during the heating process is the cleavage of the O-glycosidic bond (Fig. 3, Route 1). Some specific flavor compounds, such as geraniol and menthol, were released by the cleavage of the O-glycosidic bond^9,18^. During the glycoside pyrolysis process, different decomposition reactions usually result in different thermal decomposition intermediates^17^. As shown in Fig. 3, there are two possible pyrolytic modes (marked as Route 1 and Route 2) that produced aroma compounds for MBGL. By Route 1, the intermediates of the 4-methoxybenzyloxy group (*m/z* = 137) and glycone moiety (*m/z* = 331) can be observed in the mass spectra. Compared with Route 1, Route 2 directly results in the formation of 4-methxoybenzyl group (*m/z* = 121) and glycone moiety (*m/z* = 347).

**Figure 4:**
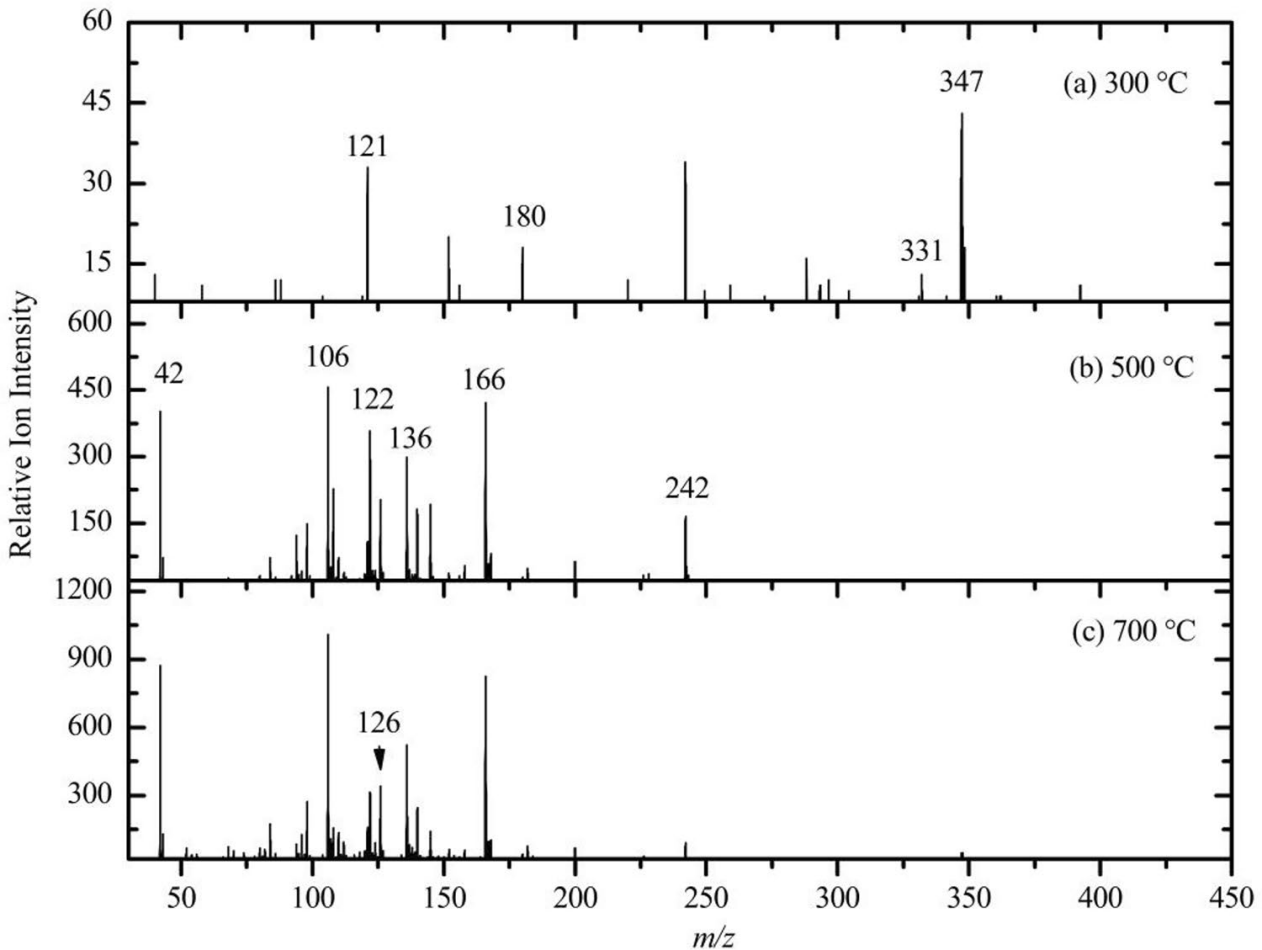
Photoionization mass spectra of the pyrolysis products of MBGL at different temperatures: (**a**) 300 °C, (**b**) 500 °C and (**c**) 700 °C.

Figure 4a shows the mass spectra of MBGL at a temperature of 300 °C with a fixed photon energy of 13.5 eV. From the picture, we can see that MBGL showed a different pyrolysis pathway than did the benzyl-2, 3, 4, 6-tetra-O-acetyl-β-d-glucopyranoside (BGL) reported in the author’s previous study^17^. The primary decomposition reaction of the BGL was the cleavage of the O-glycosidic bond (pyrolysis by Route 1) at a temperature of 300 °C. For the MBGL, Route 1 was not the principal route taken during the pyrolysis process, as the fragment ion at *m/z* 331 could not be detected. The MBGL was more likely to be decomposed via Route 2, due to the high abundance of fragment ions detected at *m/z* 347. This conclusion could also be proved by the identification of the *m/z* 121 ion in the mass spectra. The pyrolysis fragment ions at *m/z* 242 and 180 were probably produced by the loss of acetyl via the C–O bond cleavage from the MBGL. The likely reason that the MBGL showed a different pyrolysis pathway than did the BGL may be the existence of an electron-absorbing CH_3_O– group on the benzene ring.

As the temperature increased to 500 °C and 700 °C (Fig. 4b,c), in addition to the fragment ions at *m/z* 121, 242, and 347, fragment ions were produced at *m/z* 166, 136, 106, 122, and 42 at a photon energy of 13.5 eV. The *m/z* 122 ion was probably formed by the addition of hydrogen from the *m/z* 121 ion. The *m/z* 106 ion was possibly formed by the loss of methyl group from the *m/z* 121 ion. The *m/z* 242, 166, and 42 were glucose decomposition or rearrangement intermediates. These results indicate that the pyrolysis of glycosides at 300 °C was better than 500 °C and 700 °C, because there was a significant amount of flavor compound produced and fewer by-products.

## Conclusions

The thermal decomposition behaviour of a synthetic glycosidic flavor precursor was studied by TG/DTA and synchrotron VUV PIMS. TG/DTA curves showed that the temperature range of the main mass loss of the MBGL was 200–300 °C and the *T*_*p*_ of the MBGL was 246.7 °C. The pyrolysis intermediates that formed at different temperatures were identified by PIMS. Benefiting from the “soft” photoionization method and few secondary reactions at low pressure, the pyrolysis products were detected as molecular ions, M^+^. The results showed that the thermal decomposition way of MBGL was probably different from that of other glycosides. This work reported a useful application of synchrotron VUV PIMS in the thermal decomposition study of glycoside flavor precursors and demonstrated its good performance in the analysis of pyrolysis intermediates.

### Supplementary Information

Supplementary Information.

## Data Availability

The data for this study will be made available upon request from the corresponding author.
