# Supplementary material for: Thermal decomposition study of 4-methyloxybenzyl-glycoside by TG/DTA and on-line pyrolysis-photoionization mass spectrometry
Source: Sci Rep. 2024 May 24;14:11875. doi: 10.1038/s41598-024-62734-4 (PMC11126732; doi:10.1038/s41598-024-62734-4)
Supplement: Supplementary file 1 — Supplementary Information. [file 41598_2024_62734_MOESM1_ESM.docx]

**Supplementary figure**


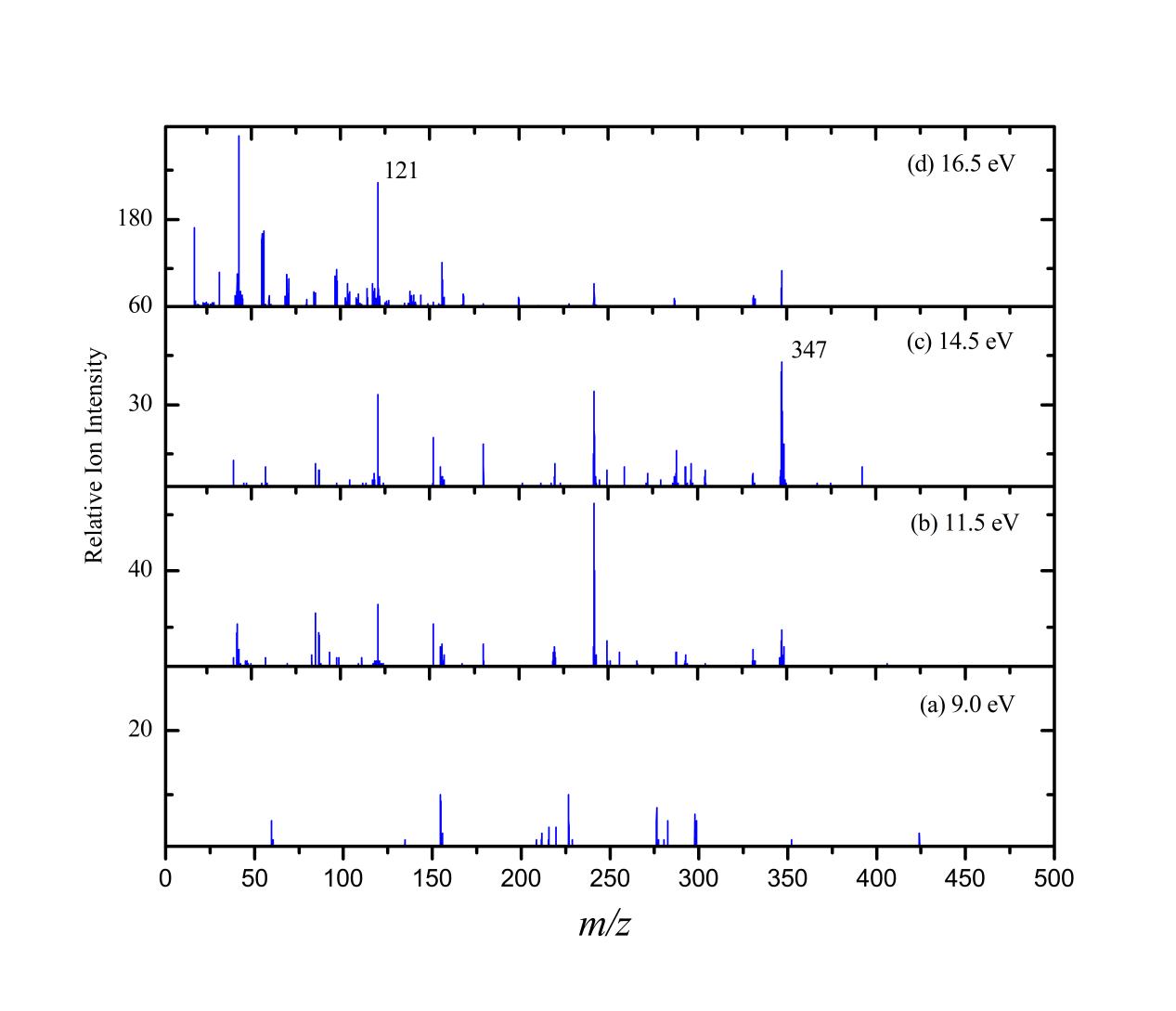


**Figure S1. (a-d)** Photoionization mass spectra of the pyrolysis products of MBGL at a fixed temperature of 300 ℃ and various photon energies: (a) 9.0 eV, (b) 11.5 eV, (c) 14.5 eV and (d) 16.5 eV.
